# Pharmacy Practice and Education in Slovenia

**DOI:** 10.3390/pharmacy7010004

**Published:** 2018-12-24

**Authors:** Borut Božič, Aleš Obreza, Jeffrey Atkinson

**Affiliations:** 1 Faculty of Pharmacy, University of Ljubljana, Aškerčeva 7, SI-1000 Ljubljana, Slovenia; Borut.bozic@ffa.uni-lj.si (B.B.); ales.obreza@ffa.uni-lj.si (A.O.); 2 Pharmacolor Consultants Nancy, 12 rue de Versigny, 54600 Villers, France

**Keywords:** pharmacy, education, practice, Slovenia, European Union

## Abstract

The PHARMINE (“*Pharmacy Education in Europe*”) project studied pharmacy practice and education in the European Union (EU) member states. The work was carried out using an electronic survey sent to chosen pharmacy representatives. The surveys of the individual member states are now being published as reference documents. This paper presents the results of the PHARMINE survey on pharmacy practice and education in Slovenia. In the light of this, we examine the harmonisation of practice and education in Slovenia with EU norms.

## 1. Introduction

The PHARMINE (“*Pharmacy Education in Europe*”) consortium surveyed pharmacy practice and education in the European Union (EU) member states, including Slovenia, between 2008 and 2011, with an update in August 2017. The methodology used and the main results obtained have been published [1]. PHARMINE gathered information on community practice, on specialised hospital and industrial practice, as well as on the mandatory education and training. PHARMINE also dealt with other personnel working in pharmacies such as pharmacists’ assistants.

PHARMINE went on to study the administrative context of pharmacy practice and education in the EU context. Like any individual country, the EU has an executive body, a legislative body and a judiciary—all features normally associated with government. Albeit, these bodies operate differently from those of an individual country. The overall governing body of the EU—the European Council—is composed of the representatives of the national governments of the member states. The EU does not govern directly the member states. It does pass legislation, but it is dependent on member states to implement it. Two aspects of EU legislation are important for pharmacy practice and education. The first concerns the free movement of people and the right of EU professionals to exercise their profession without hindrance in any member state. The second concerns the right of citizens in all member states to receive healthcare of a similar, high standard. Thus, in contrast to other parts of the world, EU pharmacy practice and education fall under two jurisdictions: EU and national. EU legisaltion is confederal in structure. Freedom of movement and of exercise of profession is the cornerstone. There is a system of automatic recognition of professional qualifications for sectoral professions such as pharmacists, as well as doctors, nurses, midwives, dentists, veterinary surgeons and architects. To work in another EU member state, professionals apply to the national authority, providing proof of the qualifications obtained in their home state. Procedures are regulated by directives issued by the European Commission of the EU. Directives are ordinances laying down the broad imperatives on practice and education [2]. A directive requires member states to achieve a particular result (e.g., harmonisation of pharmacy practice and education) but does not lay down inflexible means to obtain that result. Directives leave leeway as to the exact rules adopted. The result of this process is that member states have systems that are more or less harmonised with EU norms. 

In parallel to the above pan-national system, member states may introduce national legislation relating for example to specialised practice, and to ownership and management of pharmacies. 

Pharmacy education and training in Europe is also influenced by the Bologna agreement on the harmonization of student and staff exchange and European degree courses [3]. The Bologna agreement was signed by the education ministers of the countries of the European Higher Education Area (48 members including the 28 EU member states). It proposes recommendations that are not legally binding. These include a harmonised structure for all university degrees (including pharmacy) with a bachelor’s (3 years) followed by a master’s (2 years) degree. Here, the Bologna agreement is in opposition to the EU directive. The latter requires a five-year, “tunnel” degree structure for pharmacy, i.e., a degree course that offers no possibility for intermediate entry or exit after accomplishment of a three-year bachelor’s period. 

The idea behind the Bologna recommendations is to improve student mobility with the development of tools to promote student exchange programmes like the European Credit Transfer and Accumulation System (ECTS). This provides credits to students for defined learning outcomes. ECTS are coupled with a Diploma Supplement that describes the nature, level, context, content and status of the studies that were successfully completed by a student. This system allows students to validate studies carried out at their host university by their home university. 

This paper looks at how the EU directive and the Bologna recommendations apply in a recent member of the EU (membership in 2007), Slovenia. 

Slovenia is by its geographic position and development a part of Central Europe. It is in the group of high income countries. In order to place practice within the general health situation in Slovenia compared to Europe, it can be noted that life expectancy at birth (Table 1) in Slovenia is higher than the European average of 77 years. Healthy life expectancy (European average 68 years) is also higher. Expenditure on health is slightly lower than the European average (9.4% of GDP). Slovenia shows a tendency towards an ageing population with similar problems of health care and social care system as elsewhere in EU. Slovenia has a well distributed primary health care system with regional hospitals and two university medical centres for tertiary health care service.

## 2. Design

Information was obtained from academics and practicing pharmacists and from internet sources on:

- pharmacy;
  - practice: community, hospital and industrial;
  - legislation;
  - education and training;
- harmonisation with the EU sectoral directive on pharmacy [2] and with the Bologna recommendations [6].

Electronic survey methodology was used; data was collected in 2010 and revised in August 2017. We attempted at all times to collect objective (often numerical) data.

The information is presented in the form of tables in order to facilitate legibility. This type of presentation was developed in association with the journal’s editorial board and has been described in detail in a previous publication [7]. This format will ease the comparison of different EU countries by students and staff envisaging exchange programmes, and by researchers in pharmacy education and practice. It should be noted that this article is not intended to be a classical research manuscript but more a descriptive, qualitative research article.

## 3. Evaluation and Assessment

### 3.1. Organisation of the Activities of Pharmacists, Professional Bodies

Table 2 provides details of the numbers and activities of community pharmacists and pharmacies in Slovenia. Items are expounded in the “comments” column.

It should be noted that in law a “natural person” is an individual human being (as opposed to a legal person either private (business entity or non-governmental organization) or public (government) organization).

The number of pharmacists per population is much lower than the EU norm. Using the data in Ref. [1] and Table 2, it can be calculated that, compared to the EU linear regression estimation (for definition and calculation, see [1]), the ratio of the number of community pharmacists in Slovenia/population compared to the linear regression estimation = 0.24. The same comparison for community pharmacies produces a ratio of 0.18, again much lower than the EU norm. The number of pharmacists/pharmacy (3.4) is higher than the EU average of 2.1 ± 0.7 [1]. 

The activities and occupations of pharmacists in Slovenia are similar to those of community pharmacists in other EU member states [1]. 

Table 3 provides details of the numbers and activities of persons other than pharmacists working in pharmacies in Slovenia.

Turning to specialisation in pharmacy practice, Table 4 provides the numbers and activities of hospital pharmacists in Slovenia.

The number of pharmacists working in hospitals is again lower than the EU average. The ratio of the actual number compared to the linear regression estimation is 0.28, (for definition and calculation see Reference [1]). The ratio of hospital pharmacists is also lower (0.38). 

The duties of hospital pharmacists are similar to those elsewhere in the EU. As active members of European Association of Hospital Pharmacists (EAHP), they quickly implement the novelties into everyday practise [1,12].

Table 5 provides details of the pharmaceutical industry, industrial pharmacists, in Slovenia.

According to the 2017 data of the European Federation of Pharmaceutical Industries and Associations (EFPIA, [13]), 183 M€ are spent on R & D in Slovenia. Production represents 1354 M€ and there is a positive trade balance (net export) at 1354 M€. Slovenia ranks 9th in the EU for its positive trade balance for pharmaceuticals (it should be noted that 15/28 countries in the EU have a negative trade balance) [13].

Industrial pharmacists in Slovenia fulfil similar tasks to those elsewhere in the EU [1]. A comparison of numbers with other countries is not possible given the different job descriptions and education and training of employees in the European pharmaceutical industry.

Table 6 provides details of pharmacists working in other sectors, in Slovenia.

Table 7 provides information on professional organizations for pharmacists in Slovenia and their roles in designing conditions for pharmaceutical work. There are two types of organizations: chambers and professional associations. Both are involved in the development of the pharmaceutical profession but from different positions. Membership for both is elective, but chambers have specific authorizations by law or by Government. 

Core organizations for pharmacists in Slovenia are Slovene chamber of pharmacies and Slovenian pharmaceutical society. For professionals, working in the field of medical laboratories (laboratory medicine), including pharmacists are core organizations Chamber for laboratory medicine and Slovenian association for clinical chemistry and laboratory medicine. Main activities are shown in Table 7.

### 3.2. Pharmacy Faculties, Students, and Courses

Table 8 provides details of pharmacy higher-education institutions (HEIs), staff and students in Slovenia.

A comparison to the EU average for staff shows that Slovenia has a ratio of 1.24, for students 1.57 and for pharmacy HEIs 1.35 [1].

There are at least two levels of pharmacists’ professional orientation or specialization. One is pregraduate “in-program” by elective courses in the 3rd and 4th year of the programme, and by selection of the research topic of the master’s thesis. Table 9 provides details of past and present changes in pharmacy education and training in Slovenia.

Another is post master specialization as training at the work place combined with theoretical part, and second master degree. Table 10 below contains details of specialisations of pharmacists as postmaster training. 

Three-year specialisation programmes are completed by a terminal examination and presentation of a written work in the form of research/professional thesis. Four-year specialisation in clinical chemistry is equivalent to the EuSpLM [19] programme with a terminal examination, organized by the Chamber of Laboratory Medicine [20]. Postgraduate European Radiopharmaceutical Chemistry/Radiopharmacy Course [18], organized by the Faculty of pharmacy, University of Ljubljana [14] is recognized by the European Association of Nuclear Medicine (EANM) Radiopharmacy Committee as theoretical module 1 of the post-graduate specialization certificate program.

### 3.3. Teaching and Learning Methods

Learning methods applied in the program are expressed as contact hours as follows: lectures (48%), tutorials (12%), practical work (33%) and project work (7) (for definitions of the different methods, see [1]). Contact hours with teacher/assistant/mentor/supervisor represent approximately 40% of total study hours and traineeship 10%, which corresponds to half a year of practical work. Traineeship can be in a community or hospital pharmacy, and, for a limited number of students, a special traineeship is arranged, combining community and hospital pharmacy (there is not sufficient capacity in Slovenian hospitals for 150 students at the same time). For industry, traineeship lasts 12 months after graduation; it is not regulated by the Ministry of Health and is not part of the study.

### 3.4. Subject Areas

Table 11 provides details of student units by subject area (for definitions of the subject areas, see [1]). 

Taking the MEDISCI/CHEMSCI ratio as an indicator of the nature of the content of the M. Pharm. degree courses [22], it can be seen that the course is balanced. Other countries in the EU such as Spain also have a “balanced” course. Others have more “medical” courses such as Ireland and the Netherlands with indices of 2.6 and 1.6, respectively [22].

The course in Slovenia allows for a substantial amount of flexibility in the choice of optional and elective courses. This is shown by the variability in the ranges of minimum–maximum hours, expressed in ECTS. This variability allows the choice of a more “clinical pharmacy” course or a more “industrial pharmacy” course. This represents a certain degree of pregraduate specialisation although the nomenclature of a single “degree in pharmacy” is maintained. The variability of the subject content is also influenced by the choice of the research work for the master thesis that is defended in the 5th year but which starts already in the 1st year. 

An exact figure for generic competences is difficult to calculate as these are incorporated into many subject areas such as:

- Practical skills in practical work in laboratories,
- Language as part of pharmaceutical chemistry and other subjects where the relevant literature is in English,
- Communication as a part of social pharmacy as well as a part of the many subjects where student oral presentations are obligatory,
- First aid and communication as parts of traineeship,
- Specific field legislation is part of several courses dealing with particular regulative acts.

### 3.5. Impact of the Bologna Principles [3]

Slovenia like other EU countries has adopted the majority of the elements of the Bologna system such as the use of ECTS, double cycle (with exception for the programme of pharmacy or programmes for other regulated professions, which are integrated five-year programmes), and the Diploma Supplement. Accredited courses of life-long learning (LLL) have a similar structure (30 hrs of student workload for 1 ECTS, 11–15 hrs of which are contact, others are individual work). All programmes are accredited by NAKVIS, which is a member of European Quality Assurance Register for Higher Education (EQAR). University of Ljubljana (UL) as a whole has external quality assessment by NAKVIS. The Faculty of pharmacy is a member of European association of faculties of pharmacy (EAFP) from the very beginning in 1992 and in 2015 underwent foreign external institutional evaluation by German accreditation/evaluation agency ASSIN https://www.asiin.de/en/), a member of the EQAR at the European Association for Quality Assurance in Higher Education (ENQA). 

Student mobility is strongly supported by HEI through bilateral agreements with 53 faculties of pharmacy: 60–100 students of pharmacy are regularly involved in Erasmus and other exchange programmes every year, approximately the same number of foreign pharmacy students are hosted every year. The main obstacle for foreign students to study in Slovenia is language. All contact hours are given in Slovenian language with exception of some elective courses, which are given in English, and defined each year by the HEI. On other courses, ERASMUS and other exchange students receive English study literature and individual consultations and mentoring in English by professors. Practical laboratory work is orgamized with English-speaking subgroups when necessary (enough foreign or exchange students). For foreign students, all written and oral instructions are given in English and written and oral examinations are provided in English as well. Stuff exchanges are enabled through Erasmus plus and the Central European Exchange Program for University Studies (CEEPUS) [23].

European dimension of pharmacy study in Slovenia is further represented by:

- Common diploma in Postgraduate European Radiopharmacy Course. The postgraduate education for the title "Radiopharmaceutical Chemist/Radiopharmacist" consists of three modules in Ljubljana (Slovenia), Zurich (Switzerland) and Leipzig (Germany). The course contents follow the guidelines of the European Association of Nuclear Medicine [24].
- Summer school in Immunology in collaboration with the Institute of Pharmacology, University of Bern, Switzerland.
- The non-clinical, pharmaceutical and early clinical development of Cooperative European Medicines Development Course (CEMDC) is an integral part of the Innovative medicines initiative (IMI) Pharmaceutical Medicine Training Programme [25].

### 3.6. Impact of European Union (EU) Directive 2013/55/EC [2]

Slovenia conforms to the EU directive with a five-year integrated study of pharmacy including a half year of practice in public or hospital pharmacies (traineeship). Compared with the earlier traineeship of 12 months (after graduation), in which it was possible to include community/hospital pharmacies, laboratory medicine and industry, six months is, in the opinion of some stakeholders, too short a period for all these aspects. In addition, only pharmacies are covered by the EU directive, not industry and laboratory medicine.

The balance between theoretical and practical training is very important as:

- Regarding traineeship, some common skills can be taught at the HEI and not separately in each pharmacy.
- A university course besides providing competences for working as a pharmacist should also provide a wider education in terms of academic literacy, problem solving, etc.

## 4. Discussion and Conclusions

The pharmacy programme including a six-month training as a part of study programme is completely harmonized with EU directives for all students; although, in Slovenia, only half of graduates are employed in public and hospital pharmacies. This affects the study programme, which enables students to developed competences, in line with an EU Directive, for work also in other areas such as the pharmaceutical industry or laboratory medicine. 

It cannot be neglected that, in Slovenia, the overall number of pharmacists per population is much lower than the EU norm, the number of community pharmacists/population is lower than in EU and also the number of community pharmacies/population is much lower than the EU norm. Only the number of pharmacists/pharmacy (3.4) is higher than the EU average of 2.1 ± 0.7. Slovenian pharmacy practice also has a substantial element of public ownership. Namely, municipalities are responsible for primary health care of population including the medicine supply through pharmacies. There are 24 public institutes established by one or more municipalities, which manage 233 pharmacies (including 46 pharmacy branches). Additionally, two (public) hospital pharmacies serve as a community pharmacy as well. There are 98 private pharmacies (including 11 private pharmacy branches), all with concession. The consequence of this situation compared to that in the parts of the EU where individual ownership is the rule is that pharmacies are open also in areas where profitability is not the case (responsibility of public municipalities for balanced development in public services, demographically threatened areas). Compared to ownership by pharmacy chains, the consequence is that the spectrum of medicines of different producers is wider, independent of ownership. On the other hand, private pharmacies with concession have difficulties competing with public institutes regarding the spectrum of available medicines. 

The master curriculum is subject to regular adaptation and modification with special attention on development of students’ competences. Since with a 5-year study programme students can only develop basic competences for a novice, not for experienced professionals, continuous professional development is more and more important. The HEI together with professional associations and chambers organize yearly continuous professional development (CPD) courses, which are evaluated by the Slovene Chamber of Pharmacies and are essential for prolongation of professional licensing. More formal professional development is enabled through postgraduate specializations, which were optimized recently to fulfil the expectations of stakeholders and to be in line with modern guidelines in community, hospital and clinical pharmacy as well as in the prosperous pharmaceutical industry. 

## Figures and Tables

**Table 1:** Health statistics for Slovenia [4,5].

Total Population	2,065,879
Life expectancy at birth (years)	81
Healthy life expectancy at birth (years)	71
Total health expenditure as % of GDP	8.7

**Table 2:** Numbers and activities of Slovenian community pharmacists and pharmacies [8].

Item	Number	Comments
Community pharmacists	1144	Data: 31 December 2016. 1807 inhabitants per pharmacist; 3.4 pharmacists per pharmacy
Community pharmacies	333	Data: 31 December 2016. 6204 inhabitants per pharmacy. Branch pharmacies are included.
Competences and roles of community pharmacists		According to EU directive: Supplying prescription medicines Managing medicines for some ailments Giving advice on medicines Screening services Services to the housebound, to nursing and care homes (medication reviews, advice on storage and administration of medicines)
Ownership of a pharmacy limited to pharmacists?	Yes in the case of natural persons	70% (233) community pharmacies (pharmacy branches included) are organized as 24 public institutes established by municipalities. Private ownership of community pharmacies is limited to pharmacists or to a legal entity in which a pharmacist, who is also the manager or a member of the management body, holds more than 50% of the capital.
Are there rules governing the distribution of pharmacies?	Yes	The pharmacy network is defined on the basis of the following criteria: the needs of the population to access medicinal products and other treatment-supporting and health-promoting products, the size of the population in the catchment area of a pharmacy the road distance between pharmacies, the presence of primary-level healthcare services. The minimum distance between a new and existing pharmacy as measured by public roads shall be: at least 400 metres in urban areas, at least five kilometres in other areas. A pharmacy may be established if the number of inhabitants in the catchment area of a pharmacy exceeds 6000. A pharmacy branch shall be established in areas in which the number of inhabitants in the catchment area of a pharmacy branch exceeds 2500 and primary-level healthcare services are already in place. A stock of medicinal products may be set up by a pharmacy in a doctor’s clinic in an area at least 10 km from the nearest pharmacy or pharmacy branch. Reference [9].
Healthcare products available by other channels	Yes	Specialized shops: some are registered for over-the-counter medicines (OTC), if not they may sell only healthcare products. Some pharmacies offer an E-pharmacy service for OTC only. Reference [10].

**Table 3:** Numbers and activities of other personnel working in pharmacies in Slovenia [11].

Item	Number	Comments
Titles and number	523	Pharmacists’ assistants (pharmacy technicians). Data from 31 December 2016. There are 1.57 pharmacists’ assistants/pharmacy
Organisation providing training		Secondary school for pharmacy, cosmetics and healthcare in Ljubljana and pharmacy programs at secondary schools in Ruše and Novo Mesto
Duration of studies	4 years secondary education + 6 months’ probation	The program for pharmacy technicians comprises 4 years of secondary education (at age 15–19 years), a final exam at the end of the secondary school and 6 months’ probation in a pharmacy
Competences and roles		Supply of non-prescription medicines and non-medicinal healthcare products Storage and managing of medicines, including ex tempore preparations

**Table 4:** Numbers and activities of hospital pharmacies and pharmacists.

Item	Number	Comments
Hospital pharmacists	124	They are organized as a section of the Slovenian chamber of pharmacy [8] Data from 31 December 2016.
Hospital pharmacies	27	Data from 31 December 2016. 4.6 pharmacists per hospital pharmacy.
Competences and roles		In line with the system for the recognition of professional qualifications, EU directive 2005/36/EC [12]. In- or out-patient clinics Consultant in specialised clinical areas such as paediatrics or intensive care Part of multidisciplinary patient-care team Purchasing of drugs and medical material Monitoring of drug use Unit-dose drug distribution Production of patient-specific medicines (cytotoxic preparations, radiopharmaceuticals) Recognized competences differ from hospital to hospital.

**Table 5:** Pharmaceutical industry, and industrial pharmacists.

Item	Number	Comments
**Pharmaceutical industries**		
Companies with production, R & D and distribution	2	Two companies have R & D, production and distribution: Krka: http://www.krka.biz LEK/Sandoz: http://www.lek.si/en/ Additionally there are 9 public and 6 private companies supplying tissues and cells and medical devices: www.jazmp.si. Examples: Celica biomedical: http://celicabiomedical.com/ Educel: http://www.educell.si/en/. Blood transfusion centre: http://www.ztm.si/en/
Companies with production only	10	Galenical laboratories Example: Galex: www.galex.si
Companies with distribution and marketing only	99	99 companies are registered for the distribution of pharmaceutical and healthcare products, 3 of which are classical full-line distribution companies for pharmaceutical products. Examples: Kemofarmacija (Mckesson Europe): http://www.kemofarmacija.si/wps/wcm/connect/en/home Salus: http://www.salus.si/en Farmadent: www.farmadent.si Medis: www.medis.si/en About 80–100 pharmacists are employed in the sector of pharmaceutical distribution
Companies producing generic drugs only	3	Krka: www.krka.biz/en Galex: www.galex.si Marifarm: http://www.marifarm.si/
**Industrial pharmacists**		
Pharmacists working in industry	1100	660 pharmacists work in two main factories (Krka d.d. and LEK/Sandoz). Approximately 450 (data 2017) work in other pharmaceutical firms, mainly in pharmaco-informatics, trading and sales. A total of 8961 persons worked in the Slovenian pharmaceutical industry in 2017 [13].
Competences and roles of industrial pharmacists		Depends on position and company: R & D—drugs R & D—health care products other than drugs Synthesis and production of new chemical entities and drugs Preclinical and clinical drug evaluation (safety and efficacy) Medical devices Cosmetology Product manufacture Marketing Distribution Drug evaluation and registration

**Table 6:** Pharmacists working in other sectors.

Item		Comments
Pharmacists working in other sectors	270	50% in education 20% in laboratory medicine (clinical laboratories) 20% in administration and legislation 10% in other sectors
Sectors		Education (HEIs and secondary schools) Laboratory medicine (medical/clinical laboratories) Administration and legislation including regulators: Ministry of health; Health inspectorate; Agency for pharmaceutical products and medical devices; National institute of public healthNational Laboratory of Health, Environment and Food
Competences		Depends on sector and position. Examples: Teaching and researching Clinical evaluation of methods for analyses of biological (human) samples Interpretation of laboratory results Chemical, biological, physical analyses of medicines and medical devices Legislation about drug evaluation and registration Management

**Table 7:** Professional associations for pharmacists in Slovenia.

Item		Comments
Registration of pharmacists	Yes	According to the Law on Pharmacies (ZLD-1) [9] the Slovene Chamber of Pharmacies shall perform the following tasks (amongst others): keep the register of pharmaceutical practitioners employed with pharmacy service providers, grant, renew and revoke licences to pharmacists, keep records of compliance with the conditions for granting and renewing licences. According to the Rules on the register of providers of Laboratory Medicine (Official Gazette of RS, Nos. 113/06 and 15/17), the Slovenian Chamber of Laboratory Medicine keeps a national register of specialists in clinical chemistry/laboratory medicine and professionals with a master degree (including pharmacists) working in laboratory medicine).
Creation of pharmacies and control of territorial distribution	Yes	A new pharmacy or pharmacy branch is established after obtaining the (positive) opinion of the Slovene Chamber of Pharmacies [8] and the consent of the Ministry of Health
Ethical and other aspects of professional conduct	Yes	The codex of pharmaceutical ethics of the Slovenian Pharmaceutical Society is for members of the society only. The deontology codex in pharmacy from the Slovene chamber of pharmacies is for all pharmacists, working in pharmacy praxis. The deontology codex in laboratory medicine from the Slovenian Chamber for Laboratory Medicine is for all who work in diagnostic laboratories, including pharmacists.
Involvement in HEI courses for pharmacists		The Chamber together with the Faculty of Pharmacy organises and supervises the practical training of pharmacy students. The Chamber of Pharmacies obliges pharmacists to undergo regular continuous professional development and also organizes two-day educations for pharmacists every year.

**Table 8:** Pharmacy higher education institutions (HEIs), staff, and students in Slovenia.

Item		Comments
Number of HEIs	1	University of Ljubljana (UL), Faculty of Pharmacy [14]
Bachelor + master degrees B + M	No	Not in pharmacy where there is a fully integrated 5-year program. The faculty also teaches: B + M degrees in laboratory medicine B in Cosmetic sciences M degree in industrial pharmacy; this does not include the competencies for pharmacy practice as laid out in the EU directive [2].
Number of teaching staff (nationals)	82	Full time employment (FTE) at the faculty An additional workload (about 20 FTE) is provided by circa 100 teachers and assistants from other faculties (medicine, chemistry, mathematics and physics).
EU teaching staff	3–8 per year	As guest teachers
Non-EU teaching staff	1–3 per year	As guest teachers
Number professionals (pharmacists and others) from outside the HEI	140	Experts from the pharmaceutical industry, community pharmacies, laboratory medicine, hospitals, Ministry of health, Health Insurance Institute of Slovenia, Agency for Medicinal Products and Medical Devices of the Republic of Slovenia [15]. They are involved as: guest lecturers, mentors of practical training in pharmacies co-mentors for thesis research work
Student places	2017: 150	Based on a yearly decision of the Ministry for higher education, science and technology (*numerus clausus*).
Applicants for entry	2017: 197	Full details of student numbers are available at: (https://www.uni-lj.si/studij/prijavno_sprejemni_postopki/ (enrolment procedures) and at http://www.mizs.gov.si/fileadmin/mizs.gov.si/pageuploads/Visoko_solstvo/Statistika_in_analize/Analize_vpisa_arhiv/analiza_2017_2018.pdf (national analyses about matriculation 2017–2018)
Graduates that become registered pharmacists	2016/17: 133	Since programme was harmonized with the EU directive (extendion to 5 years, including a 6-month practice period, terminating with a state examination and thesis defence) all graduates are registered by the Ministry of health at the national register of healthcare professionals.
EU international students	125	The lectures are given in Slovenian. Exchange students receive support material and individual mentoring by professors in English. In practical laboratory work most subjects have an English-speaking sub-group when necessary (enough foreign exchange students). All written and oral instructions for foreign students are given in English. Written and oral examinations in English are provided for exchange students. Exchange students are expected to have at least an intermediate level of English knowledge.
Non-EU international students	20	The same items as is previous paragraph apply.
National entry requirements	Yes	General national secondary school examination. Graduates of some secondary technical schools (pharmacy, laboratory medicine, veterinary, cosmetics) may enter with an additional examination.
Quality assurance and validation of HEI courses	Yes	As from 2017 National agency for quality assurance in high education NAKVIS handles institutional accreditation, the first university programme accreditation (for unlimited time) and all major program changes [16]. The faculty of pharmacy collaborates with the main stakeholders: professional associations, alumni, chambers, drug producers or their associations e.g., Forum of international research & development pharmaceutical companies EIG and regulators (JAZMP)
Fees per year	0	The costs are covered from the state budget for all EU citizens and students from non-EU countries which have bilateral agreement with Slovenia. The costs are financed by government as lump sum to University, not per student.
Non-EU students	11,000 €/year	

**Table 9:** Past and present changes in education and training in Slovenian pharmacy education and training.

Item		Comments
Major changes since 1999	Yes	Harmonisation with EU directive (six-month training in pharmacy), when Slovenia entered the EU in 2004 [14]. Implementation of Bologna reform in 2007 to 2011 and later fine tuning of courses (including modifications in ECTS) with modification in examinations Orientation to competence based education and examination Introduction of radiopharmacy as national specialization Reorganization of two specializations (community and clinical pharmacy) into one.

**Table 10:** Specialisation electives in pharmacy HEIs in Slovenia.

Item		Comments
Specialized courses?	Yes	Different types of specialized courses are available: Elective courses during integrated (5 years) master program of Pharmacy Master program of Industrial pharmacy Theoretical part of postgraduate specialisation programmes is performed by HEI in cooperation with the Slovene Chamber of Pharmacies and the Slovenian Chamber for Laboratory Medicine. Such courses (but not the whole specialization) are open to graduates with a master degree in science as well as to pharmacists. Doctoral programme of Biomedicine with 11 fields [17]
Which years?	Different	Depends on programme: Electives in 3rd and 4th year and topic of master thesis in the 5th year of Pharmacy 1st and 2nd year and topic of master thesis of Industrial pharmacy 1st year of postgraduate specialization trainings. 11 fields including pharmacy, toxicology, laboratory medicine, electives in the 2nd year of doctoral study Biomedicine, the topic of doctoral thesis
Which specialisations?		Postgraduate 3 or 4 years specialization training (for details, see ref [18]): Community and clinical pharmacy (3 years) Clinical biochemistry (4 years) Pharmacognosy (3 years) Radiopharmacy (discontinuous modular type) Analysis of Pharmaceutical Products (3 years) Pharmaceutical Engineering (3 years)

**Table 11:** Student units by subject area in ECTS [21].

Year		1	2	3		4		5		Total	
Subject area				Mi	Mx	Mi	Mx	Mi	Mx	Mi	Mx
CHEMSCI		22	16	24	29	13	26	0	15	75	110
PHYS-MATH		15	6	0	0	0	0	0	15	21	36
BIOLSCI		7	7	13	18	6	16	0	15	33	63
PHARM-TECH		0	25	8	13	5	20	0	15	36	73
MEDISCI		8	6	6	11	21	36	0	15	41	76
LAWSOC		3	0	4	9	0	15	15	30	22	57
GENERIC		5	0	0	1.5	0	2.5	25	25	30	34

CHEMSOC: chemical sciences; PHYSMATH: physical and mathematical sciences; BIOLSCI: biological sciences; PHARMTECH: pharmaceutical technology; MEDISCI: medicinal sciences; LAWSOC: law and social sciences; GENERIC: generic competences. Mi: minimu. Mx: maximum.
